# Characterization of Extracellular Vesicles from Preconditioned Human Adipose-Derived Stromal/Stem Cells

**DOI:** 10.3390/ijms22062873

**Published:** 2021-03-12

**Authors:** Alec Geßner, Benjamin Koch, Kevin Klann, Dominik C. Fuhrmann, Samira Farmand, Ralf Schubert, Christian Münch, Helmut Geiger, Patrick C. Baer

**Affiliations:** 1 Division of Nephrology, Department of Internal Medicine III, University Hospital, Goethe-University, 60596 Frankfurt/M., Germany; alec.gessner48@gmail.com (A.G.); B.Koch@med.uni-frankfurt.de (B.K.); samira.farmand@web.de (S.F.); h.geiger@em.uni-frankfurt.de (H.G.); 2 Institute of Biochemistry II, Faculty of Medicine, Goethe-University, 60596 Frankfurt/M., Germany; klann@em.uni-frankfurt.de (K.K.); ch.muench@em.uni-frankfurt.de (C.M.); 3 Institute of Biochemistry I, Faculty of Medicine, Goethe-University Frankfurt, 60590 Frankfurt, Germany; fuhrmann@med.uni-frankfurt.de; 4 Division of Allergology, Pneumology and Cystic Fibrosis, Department for Children and Adolescents, University Hospital, Goethe-University, 60596 Frankfurt/M., Germany; ralf.schubert@kgu.de

**Keywords:** adipose-derived stromal/stem cells, mesenchymal stromal/stem cells, hypoxia, extracellular vesicles, proteomics, renal tubular epithelial cells

## Abstract

Cell-free therapy using extracellular vesicles (EVs) from adipose-derived mesenchymal stromal/stem cells (ASCs) seems to be a safe and effective therapeutic option to support tissue and organ regeneration. The application of EVs requires particles with a maximum regenerative capability and hypoxic culture conditions as an in vitro preconditioning regimen has been shown to alter the molecular composition of released EVs. Nevertheless, the EV cargo after hypoxic preconditioning has not yet been comprehensively examined. The aim of the present study was the characterization of EVs from hypoxic preconditioned ASCs. We investigated the EV proteome and their effects on renal tubular epithelial cells in vitro. While no effect of hypoxia was observed on the number of released EVs and their protein content, the cargo of the proteins was altered. Proteomic analysis showed 41 increased or decreased proteins, 11 in a statistically significant manner. Furthermore, the uptake of EVs in epithelial cells and a positive effect on oxidative stress in vitro were observed. In conclusion, culture of ASCs under hypoxic conditions was demonstrated to be a promising in vitro preconditioning regimen, which alters the protein cargo and increases the anti-oxidative potential of EVs. These properties may provide new potential therapeutic options for regenerative medicine.

## 1. Introduction

Extracellular vesicles (EVs) as extracellular organelles ensure intercellular communication and are associated with various physiological and pathological processes [1]. Information can be transferred to neighboring or distant cells in the form of proteins, lipids, and different RNAs. EVs can be released from numerous cells and are detectable in almost any body fluid. Depending on the parent cell, EVs display a certain diversity and individuality, especially in the composition of their surface molecules and cargo [2]. EVs are distinguished by their origin and size into three subgroups, namely, exosomes, microvesicles, and apoptotic bodies. Exosomes represent the smallest fraction of EVs in terms of size (50–150 nm) followed by microvesicles (range from 100 to 1000 nm) and the larger apoptotic bodies (over 500 nm and up to 5 µm). All these vesicles have in common a hydrophilic core enveloped by a lipid bilayer [3]. As they are the result of an invagination of endosomes in the cytoplasm, exosomes are intraluminal vesicles. In contrast, microvesicles are formed by the evagination of the outer cell membrane [4]. Although numerous studies have been performed using mesenchymal stromal/stem cells as potential therapeutics, knowledge of interactions with resident cells and how regenerative activity is achieved is currently insufficient. All types of EVs contain a complex set of information and can act in a paracrine manner as cell-free therapeutics [5].

EVs provide bioactive components from donor to recipient cells that regulate gene expression and thus alter cellular function. It is generally accepted that the main therapeutic effects are derived from the RNA cargo (e.g., miRNA), but proteins and lipids also play a role.

It is also well known that the transplantation of mesenchymal stromal/stem cells or their conditioned medium including extracellular vesicles requires cells with a maximum regenerative capability [6]. In the last decade, optimization of the beneficial effects of cell therapy has been investigated, seeking to enhance survival, engraftment, and paracrine properties of therapeutics [7]. Recent data indicate that the regenerative potential of stromal/stem cells can be boosted by in vitro pretreatment regimens (“preconditioning”) using environmental or pharmacological stimuli, thereby enhancing their therapeutic efficacy [8,9]. The paracrine profile of pretreated cells differs according to the preconditioning regimen used. The cellular responses of different preconditioning methods are complex, as they either induce or suppress various molecular signal transduction cascades. Furthermore, the pretreatment procedure affects a great number of factors rather than a single, specific molecule or protein [9]. Recent studies have hypothesized that secretion of EVs by stromal/stem cells represents one major mechanism to enhance organ regeneration and immune modulation [10]. Due to their ability to transport large cargos of proteins, lipids, and nucleic acids, which can alter the function of single or multiple target cells, EVs have been investigated extensively in recent years. Furthermore, EVs have been considered as a vehicle of active biomolecules in order to reduce inflammation or support tissue repair [11,12].

The aim of the present study was the isolation and characterization of EVs from hypoxic preconditioned adipose-derived stromal/stem cells (ASCs). For this purpose, EVs were isolated from ASCs cultured under normoxic and hypoxic environment by size exclusion chromatography and characterized by nanotracking analysis. The protein loading of the EVs was then characterized by mass spectrometry. In addition, the effects of isolated EVs on renal tubular epithelial cells were investigated.

## 2. Results

### 2.1. ASC Preconditioning and EV Isolation

**Figure 1:**
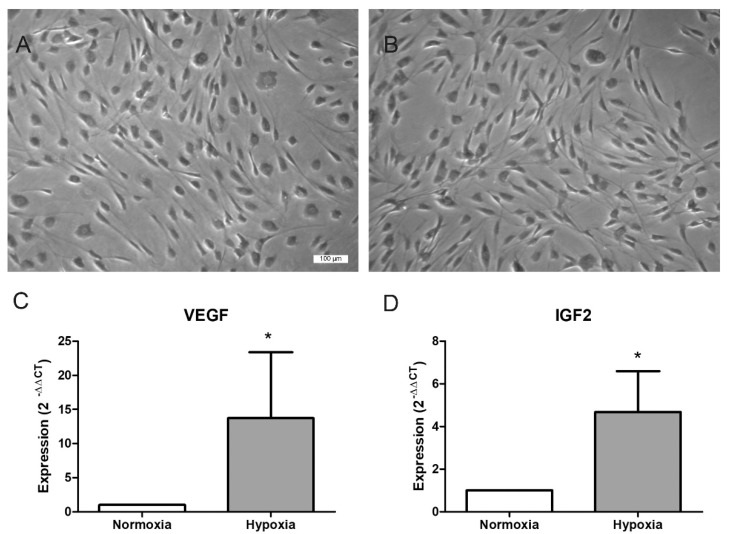
Characterization of adipose-derived mesenchymal stromal/stem cells (ASCs) in normoxia and hypoxia. (**A**,**B**) Cell morphology after culture in a normoxic (**A**) or a hypoxic environment (1% O_2_) (**B**) after 48 h (phase contrast microscopy, scale bar = 100 µm). (**C**,**D**) Effect of hypoxia on mRNA expression of vascular endothelial growth factor (VEGF) and insulin-like growth factor 2 (IGF2). Expression was quantified by qPCR analysis, normalized to β-actin, and calculated relative to the control using the ΔΔCT method (mean ± SD; *n* = 5, * *p* < 0.05).

ASCs retained their characteristic spindle-shaped fibroblastic morphology with several cell extensions, even in serum-free culture, both under normoxic (Figure 1A) and under hypoxic conditions (Figure 1B) for 48 h. Nevertheless, hypertrophy of the cells occurred in later passages and was an indication of senescence, which led to their exclusion from the experiments.

To characterize the influence of hypoxia on ASCs, gene expression of vascular endothelial growth factor (VEGF) and insulin-like growth factor 2 (IGF2) was investigated. The mRNA expression of the target genes was normalized to the housekeeping gene, β-actin. The relative expression to the control was determined as x-fold expression using the ΔΔCT method. Hypoxia significantly increased the expression of VEGF and IGF2. VEGF expression was elevated 13.7-fold (±9.6) and IGF2 expression 4.7-fold (±1.9) in ASCs cultured under hypoxic conditions compared to ASCs growing under normoxic conditions.

**Figure 2:**
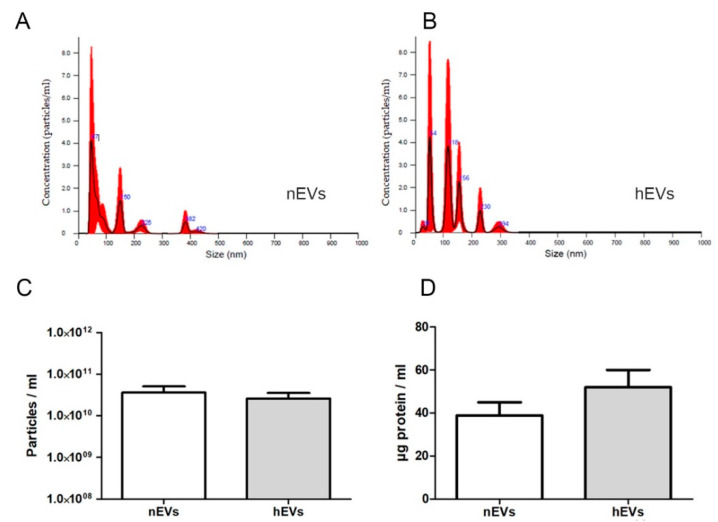
Characterization of isolated nanoparticle tracking analyses (EVs) from ASCs in normoxia and hypoxia. (**A**,**B**) Representative nanoparticle tracking analyses (NTA) of EVs isolated from ASCs after culture in a normoxic (**A**) or hypoxic (**B**) environment for 48 h. (**C**) Calculated absolute average number of isolated EVs measured by NTA using a NanoSight NS500 (*n* = 13, ASCs cultured under normoxic conditions (nEVs); *n* = 9 ASCs cultured under hypoxic conditions (hEVs)). No significant differences were detected. (**D**) Calculated average protein content of isolated EVs (µg total protein normalized to mL conditioned medium used for EV isolation, *n* = 17 (nEVs), *n* = 10 (hEVs)). No significant differences were detected.

For EV isolation, ASCs were grown to subconfluency (approximately 11,000 cells/cm^2^) under standard cell culture conditions. The cells were washed twice with phosphate-buffered saline (PBS) before the serum-containing culture medium was replaced with pure medium lacking FBS in order to assure that all EVs originated from ASCs in the following isolation process. Afterwards, cells were cultured for 48 h either under standard (normoxic, 21% O_2_) or under hypoxic (1% O_2_) conditions as a preconditioning regimen. Subsequently, conditioned medium was collected and used for further experiments and EV isolation via size exclusion chromatography (SEC). Overall, 46 cell culture media samples were gathered from nine different ASC isolations. Hypoxic preconditioning of ASCs was performed 13 times. Accordingly, 13 EV samples were isolated from the conditioned medium of ASCs cultured under hypoxic conditions (hEVs), and 33 EV samples were isolated from the conditioned medium of ASCs cultured under normoxic conditions (nEVs). After isolation, EVs were first characterized by nanoparticle tracking analysis (Figure 2A–C) and their protein content (Figure 2D), whereas not all samples collected were used for nanoparticle tracking analyses (NTA) and protein content measurements.

Examination of EV concentration and size using a NanoSight NS500 revealed an absolute average particle concentration of 3.62 × 10^10^ ± 1.45 × 10^10^ nEVs/mL isolated EV solution (*n* = 13) and 2.57 × 10^10^ ± 0.97 × 10^10^ nEVs/mL isolated EV solution (*n* = 9), respectively (Figure 2C). Normalized to mL of ASC culture supernatant used for EV isolation, this corresponds to an average of 2.15 × 10^9^ nEVs/mL and 1.76 × 10^9^ nEVs/mL culture supernatant, respectively (conditioned medium). The average size of isolated EVs ranged between 30 and 450 nm, with a mean of 168 ± 24 nm in nEV isolations, and a mean of 167 ± 17 nm in hEV isolations. In the measurements, no particles bigger than 500 nm were detected.

The average protein concentration in isolated hEVs was higher than that in nEVs. The average protein concentration of the EV isolations was 38.9 ± 6.1 µg/mL (nEVs) and 52.0 ± 8.0 µg/mL (hEVs), respectively (normalized to mL conditioned medium used for EV isolation) (Figure 2D). The average absolute protein concentration in the EV isolations was 260 µg for nEVs and 317 µg for hEVs (in 400 µL EV solution after SEC and final concentration of the solution). No significant differences were detected in the particle size and protein content analyses.

### 2.2. Proteomics

**Figure 3:**
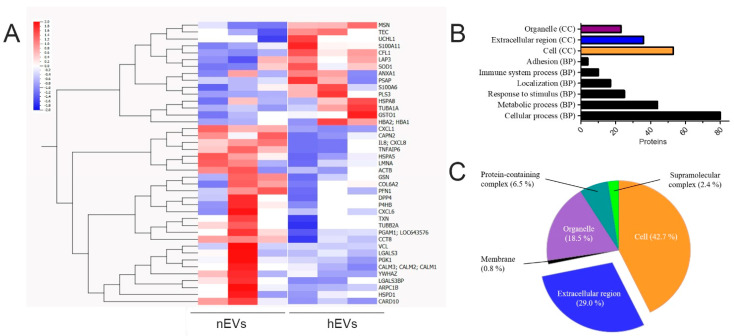
Analysis of proteomics. Six selected protein samples from EV isolations (3 nEVs versus 3 hEVs samples) were used to perform proteomic analysis via mass spectrometry. (**A**) Heatmap with hierarchical clustering. Forty-one human proteins were detected with different cargos between nEVs and hEVs (either >1.5 or <0.6 in the ratio hEVs/nEVs). (**B**,**C**) Proteomic analysis of hEVs. (**B**) Bar chart displaying the number of proteins that cluster for selected gene ontology (GO) terms for the cellular component (CC) and biological process (BP). (**C**) Pie chart showing the percentage of detected proteins that cluster for indicated cellular components (CC GO terms).

The protein cargos of EVs obtained from hypoxic preconditioned ASCs were compared to EVs derived from cells cultured under normal cell culture conditions. Therefore, six selected protein samples from EV isolations (three nEVs versus three hEVs samples) were used to perform mass spectrometry (MS). We were able to detect 139 proteins in nEVs and hEVs (Table S1). Sixteen of these proteins were identical in the combined top 30 hits from other studies with EVs from mesenchymal stromal/stem cells [13]. The evaluation of all detected proteins identified 41 human proteins, for which different loading could be detected between nEVs and hEVs (either >1.5 or <0.6 in the ratio hEVs/nEVs) (Figure 3A).

In order to understand the biological background of the detected proteins in hEVs, the gene ontology (GO) terms of the bioinformatic analysis tool PANTHER were used to cluster the identified proteins regarding their biological process (BP) and cellular compartment (CC) (Figure 3B,C). The bar chart (Figure 3B) represent the number of identified proteins in hEVs that clustered for the respective GO term. Most of the proteins clustered for the GO term “cellular process”, which also includes intercellular communication. Furthermore, many proteins are associated with the terms “metabolic process” and “response to stimulus”. Regarding the pie chart that illustrated GO terms for CC (Figure 3C), proteins clustering for the term “cell” were predominant, and the terms “organelle” (18.5%) and “extracellular region” (29%) were also highly increased.

The most highly and significantly altered protein, cofilin 1 (CFL1), which plays a major role in cellular organization and regulation of apoptosis, was increased more than four-fold compared to the control (calculated as ratio hEVs/nEVs). In contrast, the cargo of the proteins interleukin-8 and tumor necrosis factor-inducible gene 6 protein (TNFAIP6) was significantly reduced in hEVs (0.157 and 0.181, respectively). Nevertheless, only 11 statistically significant hits with *p* < 0.05 were identified (Table 1). The complete list of the 139 detected proteins and their respective changes compared to the normoxic control are listed in the Supplementary Materials (Table S1). An additional 22 identified proteins could not be quantified and were therefore not included in the tables.

### 2.3. Effects of EVs on Epithelial Cells

**Figure 4:**
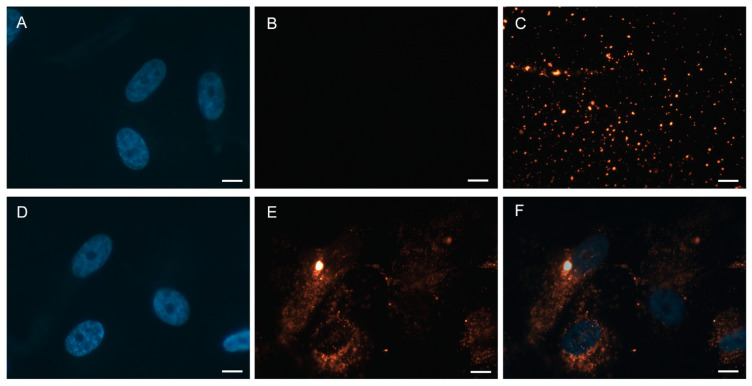
PKH26 staining of EVs and incorporation into cultured tubular epithelial cells (TECs). Fluorescence microscopy showed that TECs (nuclei stained with 4′,6-diamidin-2-phenylindol (DAPI)) uptake PKH26-labeled EVs (red). (**A**,**B**) Negative control. TECs incubated with a processed PKH26 solution without EVs ((**A**) nuclei staining, (**B**) red fluorescence channel). (**C**) PKH26-labeled EVs on an adhesion slide. (**D**–**F**) TECs incubated with PKH26-stained EVs clearly showing incorporation of labeled EVs into the cytosol of TECs ((**D**) nuclei staining, (**E**) red fluorescence channel, (**F**) overlay of (**D**) and (**E**)). Scale bar = 10 µm.

First, the uptake of PKH-stained EVs in tubular epithelial cells (TECs) was visualized by immunofluorescence microscopy (Figure 4). Therefore, isolated EVs were incubated with the red-fluorescent dye PKH26, which incorporates its long-chain alkyl tails into the membrane bilayer. Unbound dye in the solution was then removed by a second SEC. Performing this SEC after EV labeling was mandatory to remove the unbound dye and thus reduce the background of the staining. As a control, SEC was also performed with a PKH26 solution in the absence of EVs (processed PKH26 solution) (Figure 4A,B). Labeled EVs or the control were added to TECs cultured in a chamber slide and incubated for 24 h. TECs were fixed and nuclei were counterstained with 4′,6-diamidin-2-phenylindol (DAPI). Moreover, PKH26-labeled EVs were examined on an adhesion slide without cells (Figure 4C).

The processed PKH26 solution as a negative control showed no signal for the red dye, indicating that unbound PKH26 was removed efficiently by SEC (Figure 4A,B). In contrast, numerous particles were observed when labeled EVs were bound on an adhesion slide without cells (Figure 4C) or incubated on a chamber slide with TECs (Figure 4D–F). It was revealed that EVs were incorporated by TECs and distributed all over the cytosol by sparing the nucleus (Figure 4E,F).

**Figure 5:**
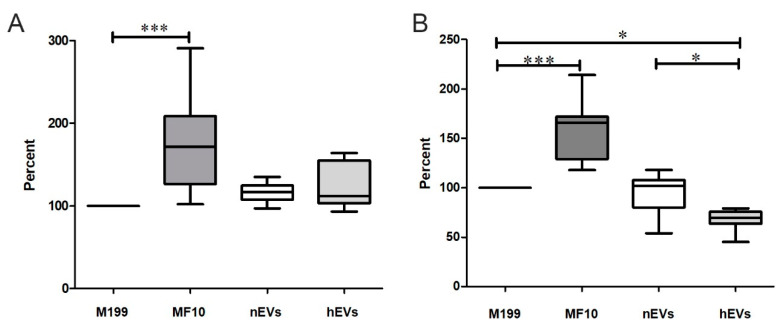
Effect of nEVs and hEVs on cultured TECs. (**A**) Cell viability after treatment with EVs. TECs were cultured with serum-free medium (M199), standard cell culture medium containing 10% fetal bovine serum (FBS) (MF10), or M199 containing nEVs or hEVs (3 × 10^8^ EVs/well, correlating to an estimated 80,000 EV-releasing ASCs) for 48 h. The 2,3-bis-(2-methoxy-4-nitro-5-sulfophenyl)-2*H*-tetrazolium-5-carboxanilide (XTT) assay was performed, and optical density was measured in a microplate reader at 492 vs. 650 nm (arbitrary units). Results were calculated as percent versus serum-free M199 as a control (=100%) (mean ± SD, *n* = 12–16). (**B**) Measurement of oxidative stress using 2′,7′-dichlorofluorescein diacetate (DCF-DA). TECs were cultured with serum-free medium (M199), standard cell culture medium containing 10% FBS (MF10), or M199 containing nEVs or hEVs (3 × 10^8^ EVs/well, correlating to an estimated 80,000 EV-releasing ASCs) for 48 h. Then, DCF-DA was added for 30 min at 37 °C. Fluorescence of intracellular DCF was measured and normalized to serum-free M199 (=100%; mean ± SD, *n* = 7–8). *** *p* < 0.001, * *p* < 0.05.

The effects of EVs isolated from ASCs cultured under normoxia or hypoxia on epithelial cells were then tested by a viability assay and the measurement of cellular reactive oxygen species (ROS) after incubation with nEVs and hEVs. The metabolic activity, which refers to cell viability and proliferation ability, was examined by performing a 2,3-bis-(2-methoxy-4-nitro-5-sulfophenyl)-2*H*-tetrazolium-5-carboxanilide (XTT) assay. The measured values were normalized to the serum-free M199 (control) as the purified EVs were also diluted in serum-free M199. Serum-containing medium (MF) served as a positive control. MF10 significantly increased cell viability (Figure 5A). Compared to the control, no other treatment displayed a significant effect on the cell viability. It can be stated that neither nEV nor hEV had a significant effect on viability (Figure 5A). On the other hand, a significant anti-oxidative effect of hypoxic preconditioned EVs was detected. Incubation of TECs with hEVs for 48 h resulted in a significant reduction in cellular ROS compared to the control (M199) and to nEVs, respectively (Figure 5B). After TECs were incubated with hEVs for at least 48 h, ROS levels strongly decreased (67.5 ± 10.6%). Culture in serum-containing MF10 significantly increased the intracellular ROS level (158.7 ± 32.2%). In summary, it can be stated that hypoxic preconditioning led to a stronger anti-oxidative effect of EVs.

## 3. Discussion

Cell-based therapies in the context of regenerative medicine for organ or tissue repair are often associated with the use of mesenchymal stromal/stem cells and especially adipose-derived stromal/stem cells. Human lipoaspirate serves as an ideal source for harvesting ASCs as it yields a sufficient amount of multipotent cells and is easily accessible. Due to their capability of releasing trophic factors including extracellular vesicles that act in a paracrine manner, ASCs are of great importance in biomedical research. Furthermore, the secretome of ASCs can be modified by in vitro preconditioning regimens such as a hypoxic microenvironment. Hypoxia also seems to alter the amount and cargo of released EVs and thereby enhance regeneration promoting, immunomodulatory, and anti-apoptotic potential [6,8,14,15]. Currently, the EV cargo after preconditioning, e.g., cultivation under hypoxic conditions, is not comprehensively characterized.

Culture of mesenchymal stromal/stem cells under a hypoxic pretreatment regimen has been shown to maintain stemness of the cells and increase their survival and proliferation by activation of anti-apoptotic signaling [16]. Hypoxic conditions lead to the stabilization of hypoxia-inducible factor-1α, which is then translocated to the cell nucleus, and induction of the expression of proangiogenic genes, e.g., VEGF [17,18]. In addition, the induction of IGF2 as a major growth factor and anti-apoptotic signaling molecule has been demonstrated in hypoxia [9]. In the present study, the expressions of IGF2 and VEGF have been shown to be enhanced as a cellular response to hypoxia. Nevertheless, as cell-based therapy is linked to safety risks, the application of conditioned medium or purified EVs of preconditioned ASCs might be a safer approach.

With regard to the isolation method of EVs from the cell culture supernatant, various methods have been described in recent years that make it difficult to compare the published results of the various studies. It was observed that differential ultracentrifugation and size exclusion chromatography are commonly used. In this work, EVs were isolated by separating the conditioned medium from ASCs based on size difference via SEC using Sepharose columns [19]. Preceding filtering and concentration steps were necessary in order to decrease the volume and to remove cell contaminations and apoptotic bodies. The efficacy and reproducibility of this method, which also preserves the vesicular structure and content, has been proven recently [19,20]. By performing nanoparticle tracking analysis, it was confirmed that numerous molecules with a size between 30 and 450 nm were isolated. Particles with a size > 500 nm, including small apoptotic bodies and large microvesicles, have not been detected in nEV and hEV preparations. In this context, however, it should also be mentioned that we filtered the harvested conditioned medium through a 0.45 µm filter and thus not only apoptotic vesicles but also larger EVs were eliminated. A follow-up study comparing the EV purification with complete conditioned medium would help to determine the effects of larger vesicles and other constituents of the conditioned medium. Remarkably, no increase in EV concentration after hypoxic preconditioning was detected. In our study, SEC with ASC supernatant yielded an adequate number of functional EVs free from contaminations and apoptotic bodies.

Regarding their function, recent studies have revealed that EVs act as a vehicle of intercellular communication via a heterogeneous group of coding and non-coding RNA molecules and proteins. However, EVs have also been shown to contain predominantly miRNAs < 300 nucleotides in size as well as intact and fragments of mRNA and (long) non-coding RNA. While it is scientifically accepted that the exchange of these different RNA molecules plays the main role in the therapeutic effect of EV, proteins and lipids are also transferred into the target cells. Therefore, a limitation of the current study is the lack of data on EV RNA loading, which needs to be elucidated in another study.

In the current study, we analyzed the protein cargo in SEC-isolated EVs and compared the influence of a normoxic pretreatment to a hypoxic pretreatment on this protein cargo. The proteome of EVs has been reported manifold [21], and a top hit list of the identified proteins has been published [13]. We found 16 of the described 30 most detected EV proteins in our EV isolations [13]. Analyzing the proteome for the involvement in biological processes (BP) indicated an important role of EVs in key cellular processes [22]. The global proteomic analysis in this study is in line with these findings. The identification of EV protein cargo by mass spectrometry validated that most proteins are part of key biological processes, such as cellular communication, immune modulation, and metabolism. The association of proteins with the GO term “response to stimulus” indicates the induction of protein loading into EVs upon hypoxic preconditioning. For example, a prominent increase for cofilin 1 (CFL1) was detected. CFL1 is involved in cytoskeleton organization, is highly susceptible to the intracellular redox status, and is associated with cell survival and T-cell activation [23]. Therefore, increased cargo of CFL1 may possess an advantageous effect on damaged epithelia and should be further investigated in future projects.

Using an in vitro model of cell regeneration, the effects of preconditioned EVs on the viability and intracellular ROS levels of injured epithelial cells were investigated. Therefore, subconfluent renal tubular epithelial cells were used, as they share several characteristics with wounded epithelial cells [24]. It was proven that cultured TECs incorporate EVs by staining them with PKH26 dye and analyzing their uptake. Labeling with PKH26 allows visualization of EVs via fluorescence microscopy, but unbound dye can aggregate and produce false-positive signals [25]. It has been shown that size exclusion chromatography using Sepharose CL-2B is sufficient to remove excessive dye after labeling of EVs. Similar results have been reported for CFSE staining of EVs with subsequent SEC in order to reduce background and false-positive signals [26]. Even though the uptake of EVs by TECs was demonstrated, the result of the viability assay demonstrated that the cell viability and corresponding cell proliferation did not increase significantly after TECs were treated with either nEVs or hEVs. A different picture is given by the measurement of oxidative stress. A significant decrease in cellular oxidative stress could be proven when TECs were treated with hEVs. This is in line with previous findings, as it has been shown that EVs induce several oxidation-reducing processes in vivo upon hypoxic preconditioning [27]. As ROS is a key player in mediating physiological and pathophysiological processes, the ROS-reducing activity of hEVs is of key interest in the context of inflammation and cancer [28,29]. Furthermore, in an ischemia/reperfusion model of myocardial infarction, EVs improved cardiac function significantly by decreasing apoptosis, oxidative stress, and inflammation [30]. As an underlying mechanism, it has been shown that peroxiredoxins (PRDX) and glutathione S-transferase (GST) transferred by EVs reduced intracellular stress [31]. In the present work, both proteins were detected in the EVs. We found GST omega 1 (P78417), an anti-oxidant enzyme, to be enhanced in EVs after hypoxic preconditioning. In addition, superoxide dismutase-1 (SOD1, P-00441), an isozyme responsible for destroying free superoxide radicals, was also found to be enriched in hEVs, but the enhanced cargo of both proteins into hEVs was not present in a statistically significant manner. The cargo of PRDX into hEVs was not enhanced. Nevertheless, it can be concluded that the regulation of intracellular ROS levels using ASC-derived EVs unlocks great potential in the setting of regenerative therapies, which can be further boosted by hypoxia as an in vitro preconditioning regimen.

In summary, the present work characterized the cargo and properties of EVs derived from hypoxic preconditioned ASCs. It was clearly shown that this in vitro pretreatment affected ASC EVs, resulting in an altered EV protein cargo but only with minor changes. Furthermore, using in vitro assays, the anti-oxidative effect of hypoxic preconditioned EVs on renal tubular epithelial cells was clearly demonstrated. Using EVs as paracrine effectors in cell-free regeneration-promoting therapies may reduce the risk of side effects, but this approach demands vesicles with a maximum regenerative capability. Culturing ASCs under hypoxic conditions was shown to be a promising in vitro preconditioning regimen, increasing the anti-oxidative effect of isolated EVs. These properties provide new potential therapeutic options for diseases that have limited, mostly supportive treatment options. In conclusion, EVs isolated from hypoxic preconditioned cells represent a particularly promising approach to regenerative medicine.

## 4. Materials and Methods

### 4.1. Cell Isolation, Culture, and Characterization

Human adipose-derived stromal/stem cells (ASCs) were isolated from adipose tissue obtained from nine female donors undergoing cosmetic liposuction. Aspirated tissue was digested at 37 °C with 0.075% collagenase I (CellSystems, Troisdorf, Germany) under continuous agitation for 45–60 min. The stromal–vascular fraction was separated from the remaining fibrous material and the floating adipocytes by centrifugation at 300× *g*. The sedimented cells were washed with phosphate-buffered saline (PBS) and filtered through a 100 µm pore filter (Millipore, Schwalbach, Germany). Erythrocyte contamination was reduced by density gradient centrifugation (Bicoll; Biochrom, Berlin, Germany). Then, cells were plated for initial cell culture and cultured at 37 °C in an atmosphere of 5% CO_2_ in humid air (Normoxia). Primary cell isolates and cultured cells were fully characterized as described previously [32,33]. Dulbecco’s modified Eagle’s medium (DMEM; Sigma, Taufkirchen, Germany) was used with a physiologic glucose concentration (100 mg/dL) supplemented with 10% fetal bovine serum (FBS; No. S0615, Lot 0001640839, Sigma/Merck, Darmstadt, Germany) as the culture medium. The medium was replaced every three days. Cells were passaged at 85–90% confluency by trypsinization. The first–fourth passage of ASC was used for the experiments. Cell morphology was examined by phase contrast microscopy. Expression of characteristic markers was proven by flow cytometric analysis of CD73, CD90, and CD105 expression, as shown in the Supplementary Materials S1a and described previously [33]. Tri-lineage differentiation potential of cultured ASC was proven by specific media, as shown in the Supplementary Materials S1b and described previously [34,35].

Human renal tubular epithelial cells (TECs) were isolated from donors undergoing tumor nephrectomies, as previously described [36]. Cells were prepared from parts of the kidney that were not involved in renal cell carcinoma. In brief, the tissue was sliced and digested with collagenase/dispase. Tissue was then passed through a 106 µm mesh and incubated with collagenase, DNase, and MgCl_2_. Then, Percoll density gradient centrifugation was performed, and the isolated cell fraction was seeded in culture flasks. Cells were cultured in medium 199 supplemented with 10% FBS at 37 °C and 5% CO_2_ under humidified conditions. For the first 2–3 days after isolation, the culture medium was further supplemented with the antibiotic meropenem (100 µg/mL). Cells were passaged by trypsinization. Primary isolated and cultured cells at passages 2–5, which is equivalent to approximately 15–35 days in culture, were used for the experiments.

### 4.2. Preconditioning of ASCs

ASCs were either cultured under standard conditions (controls in normoxia, 21% O_2_) or preconditioned by incubation in a hypoxic environment (1% O_2_) [14]. For this purpose, cells were grown to subconfluency and were washed twice with PBS. Cells received fresh serum-free low-glucose DMEM without supplements and were then placed in an InvivO_2_ 400 at 1% oxygen for 48 h. Normoxic controls were also cultured in serum-free low-glucose DMEM without supplements and were placed in a Hera cell incubator at 21% oxygen. After 48 h of preconditioning, cells and preconditioned medium were collected. To characterize the effect of the hypoxic pretreatment on ASCs, we analyzed VEGF and IGF2 mRNA expression by quantitative PCR analysis and made calculations using the ∆∆CT method [37]. Levels of target gene expression were estimated by 2^−∆∆Ct^ (detailed method and primer list in the Supplementary Materials S2).

### 4.3. Isolation of Extracellular Vesicles

Preconditioned medium (PCM) was used to isolate EVs from normoxic (nEVs) or hypoxic (hEVs) pretreatment for 48 h (growth area 150 cm^2^ with 16 mL serum-free DMEM). After 48 h incubation, PCM was centrifuged for 10 min at 600× *g* in order to remove cell debris. PCM was then filtered using a 0.45 µm PVDF filter and concentrated 10-fold by centrifugation at 1500× *g* for 20 min using 30 kDa molecular weight cut-off Centriprep 30 K filters. The PCM was then either stored at 4 °C for further analysis or used for isolation of extracellular vesicles (EVs). EVs were isolated from PCM by size exclusion chromatography (SEC) using Sepharose CL-2B columns [19,38]. The PCM was applied onto a Sepharose CL-2B column, which was washed and equilibrated with PBS. As elution buffer, PBS was applied to the column until 18 flow-through fractions with a respective volume of 500 µL were collected [39]. The fractions 7–12 containing extracellular vesicles were pooled and subsequently concentrated (to approximately 400 µL EV solution) using a 3 kDa molecular weight cut-off Amicon filter by centrifuging for 25 min at 2800× *g* [19]. EV samples were either used immediately or stored at −80 °C for further characterization.

### 4.4. Characterization of Extracellular Vesicles

Nanoparticle tracking analysis (NTA) was used to determine size distribution and concentration of the isolated EVs [40]. A total of 10 µL of concentrated EV solution (nEVs and hEVs) was diluted 1:100 with nuclease-free water and analyzed using a NanoSight NS500 (Malvern Panalytical, Malvern, UK) according to the manufacturer. EVs were illuminated with a laser, and the scattered light was captured using a camera with a 20-fold microscopic magnification, which allows the analysis of particles with a diameter of 10–1000 nm. Then, the software calculated the hydrodynamic diameter of particles [41]. Across all measurements, camera settings were fixed with a camera level of 14 and a camera gain of 1.5. The temperature was set to 28 °C. Six videos of with a duration of 30 s were captured for each sample and analyzed with the Nano Sight NTA 3.2 software, setting the threshold to 14 and the gain to 1.5.

Additionally, protein concentration of the EV isolations was measured. Therefore, 10x RIPA buffer (Cell Signaling, Frankfurt/M., Germany) was added to EV solutions and incubated on ice for 30 min in order to extract proteins. The lysate was centrifuged for 10 min at 10,000× *g* and 4 °C, and the supernatant containing proteins was transferred to a 1.5 mL tube. Protein concentration was determined using a commercial assay according to the protocol of the manufacturer (DC Protein Assay, Bio-Rad, Feldkirchen, Germany). Total protein content of the samples was calculated using a protein standard curve with bovine serum albumin and was then normalized to mL conditioned medium used for EV isolation.

In selected samples, an additional characterization of SEC-isolated EVs was conducted by Western blotting for CD44, CD63, and b-actin (Supplementary Materials Figure S2).

### 4.5. Proteomics

For mass spectrometry (MS), samples were precipitated with trichloroacetic acid and digested with Lys-C and trypsin. Then, proteins were labeled with a tandem mass tag (1 µL reagent per sample), pooled, and purified by SDB-RPS stage-tip cleanup. For liquid chromatography–mass spectrometry, samples were separated on an Easy-nLC 1200 and subsequently sprayed into a QExactive HF mass spectrometer equipped with a nanoFlex ion source. Full scan MS spectra (350–1400 *m*/*z*) were obtained, and the 20 most intense peptides were isolated and fragmented by higher-energy collisional dissociation. Then, tandem mass spectrometry (MS/MS) spectra were obtained. Raw data were analyzed using Proteome Discoverer 2.4 software by searching against the trypsin digested Homo sapiens UniProt database (TaxID: 9606). For further processing, results were exported to excel files. Gene ontology (GO) term enrichment analysis was performed with DAVID (Version 6.8) and PANTHER (**P**rotein **AN**alysis **TH**rough **E**volutionary **R**elationships, Version 14.1) [42,43].

### 4.6. PKH26 Staining of EVs on Epithelial Cells

Isolated EVs were stained with a commercially available PKH26 red fluorescent labeling (Merck, Darmstadt, Germany). A 200 µL concentrated EV solution was diluted in 300 µL Dilution C before 500 µL 15 µM PKH26 solution was added. After incubation at RT for 3 min, the mixture was applied onto a Sepharose CL-2B column, and SEC was performed in order to remove unbound dye [26]. As a control, SEC was also performed with a PKH26 solution in the absence of EVs (processed PKH26 solution). The isolated EV solution was concentrated using a 3 kDa molecular weight cut-off Amicon filter by centrifuging for 20 min at 2800× *g*. Subsequently, a 60 µL EV solution stained with PKH26 was added to 340 µL M199 cell culture medium with 10% FCS, added to cultured TECs in an 8-well chamber slide and incubated at 37 °C and 5% CO_2_ under humified conditions for 24 h. Then, the medium was removed, and cells were washed with PBS and fixed with 4% paraformaldehyde. For staining of cell nuclei 2 mM 4′,6-diamidin-2-phenylindol (DAPI) was used. Finally, the slide was covered with Mowiol and a coverslip. In order to analyze PKH26-stained EVs without contact to TECs, a 10 µL stained EV solution was pipetted onto a well of a washed adhesion slide (Paul Marienfeld, Lauda-Königshofen, Germany). The remaining solution was removed after incubation at RT for 10 min, and the slide was covered with 35 µL Mowiol and a coverslip. All slides were stored overnight at 4 °C before pictures were taken with a fluorescence microscope (Axioplan; Carl Zeiss AG, Oberkochen, Germany).

### 4.7. Effect of EVs on Epithelial Cells

To show the effects of EVs isolated from ASCs cultured under normoxia or hypoxia on epithelial cells, we tested the viability of TECs and cellular reactive oxygen species (ROS) after incubation with nEVs and hEVs. TECs were seeded in 96-well cell culture plates at a density of 5000 cells/well and incubated overnight under humidified conditions at 37 °C and 5% CO_2_. The next day, cells were treated with nEVs or hEVs (diluted 1:10 in serum-free M199 medium) and incubated at 37 °C and 5% CO_2_ for 48 h. As controls, cells were cultured with serum-free M199 medium as a negative control as well as with serum-containing M199 medium as a positive control for 48 h.

Then, a metabolic cell assay using XTT was performed according to the manufacturer’s protocol (AppliChem, Darmstadt, Germany). Finally, the absorbance of the samples was measured at a wavelength of 490 vs. 650 nm with a spectrophotometer, and the cell viability as a function of cell number was calculated. The background signals were subtracted, and the results were normalized to serum-free M199 (=100%).

The anti-oxidative effect of the treatment was determined by the measurement of intracellular ROS levels using the fluorogenic dye 2′,7′-dichlorofluorescein diacetate (DCF-DA). TECs were incubated with EVs or controls for 48 h. Then, cells were washed twice and subsequently stained with 20 µM DCF-DA. Cells were incubated at 37 °C and 5% CO_2_ for 30 min and washed. Intracellular DCF fluorescence was measured immediately using a fluorescence plate reader (FluoStar, BMG Labtech, Ortenberg, Germany) with excitation and emission wavelengths of 485 and 538 nm. The background signals were subtracted, and the results were normalized to serum-free M199 (=100%).

### 4.8. Statistical Analysis

The statistical analysis of the measured data as well as their graphic representation were performed with the software GraphPad Prism 5.0. The presented data were calculated as mean ± standard deviation (SD). Gaussian distribution of the data was confirmed with a Shapiro–Wilk test. For two groups, differences among the mean values were statistically analyzed using Student’s *t*-test. An analysis of variance (ANOVA) was performed for several independent, normally distributed groups. In the case of equally distributed variances, one-way ANOVA with Tukey’s multiple comparison test was used. Results with *p* < 0.05 were assigned significant (* *p* < 0.05; ** *p* < 0.01; *** *p* < 0.001).

## Figures and Tables

**Table 1:** Proteomic analysis. Statistically significant hits (*p* < 0.05) with a ratio hEVs/nEVs > 1.5 or <0.6.

Gene Symbol	Accession	Ratio (hEVs/nEVs)	*p* Value
CFL1	P23528	4.642	0.011
TEC	P42680	3.066	0.044
PLS3	P13797	3.018	0.015
MSN	P26038	2.070	0.010
CARD10	Q9BWT7	0.570	0.044
CAPN2	P17655	0.511	0.048
HSPA5	P11021	0.503	0.036
CCT8	P50990	0.412	0.023
CXCL1	P09341	0.255	0.001
TNFAIP6	P98066	0.181	0.012
IL8 (CXCL8)	P10145	0.157	0.006

## Data Availability

Data are available via ProteomeXchange with identifier PXD024044.

## Supplementary Materials

The following are available online at https://www.mdpi.com/1422-0067/22/6/2873/s1.

## Abbreviations

ASCs	Adipose-derived stromal/stem cells
DCF-DA	2′,7′-dichlorofluorescein diacetate
DCF	2′,7′-dichlorofluorescein
EVs	Extracellular vesicles
DAPI	4′,6-diamidin-2-phenylindol
FBS	Fetal bovine serum
IGF2	Insulin-like growth factor-2
M199	Medium 199 with physiologic glucose content (100 mg/dL)
MF10	Medium 199 supplemented with 10% FBS
PCR	Polymerase chain reaction
SEC	Size exclusion chromatography
TECs	Renal tubular epithelial cells
VEGF	Vascular endothelial growth factor
XTT	2,3-bis-(2-methoxy-4-nitro-5-sulfophenyl)-2*H*-tetrazolium-5-carboxanilide
