# Supplementary material for: Characterization of Extracellular Vesicles from Preconditioned Human Adipose-Derived Stromal/Stem Cells
Source: Int J Mol Sci. 2021 Mar 12;22(6):2873. doi: 10.3390/ijms22062873 (PMC7999156; doi:10.3390/ijms22062873)
Supplement: Supplementary file 1 [file ijms-22-02873-s001.pdf]

SUPPLEMENT

S1 *Characterization of ASCs*

S1a Flow cytometric analysis

Flow cytometric analysis was performed to characterize the specific marker expression of cultured ASCs. Cells were detached from the cell culture plastic, washed and stained with 5µl directly labeled antibodies (CD105-APC (Clone MEM226, No. 21271056, Immuntools, Friesoythe, Germany, CD90-APC (Clone 5E10, No. 21270906, Immuntools, Friesoythe, Germany, CD73-PerCP-eFluo710 (Clone AD2, No. 46-0739, eBioscience, San Diego, CA, USA), or CD45-PE (Clone MEM-28, No. 21270454, Immunotools, Friesoythe, Germany)). Labeled ASCs were then measured using a flow cytometer (BD Biosciences, Heidelberg, Germany). ASCs were gated by forward and sideward scatter to eliminate debris. The experiments included negative controls with corresponding isotype controls.

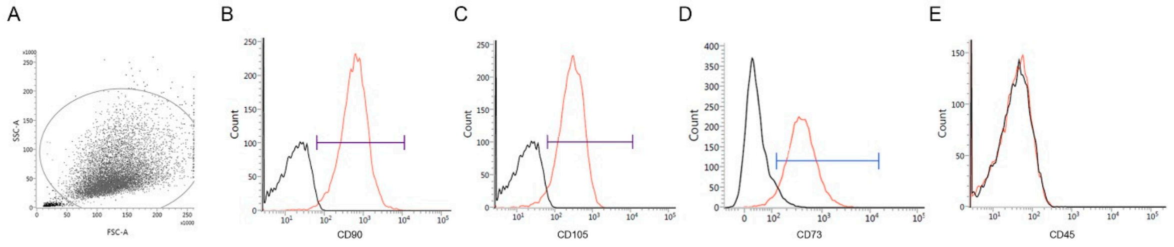

Fig. S1a: (A) Dot plot shows the forward and sideward scatter analysis with the gating strategy to eliminate debris. (B-E) Representative flow cytometric overlay histograms of characteristic marker expression (CD90 (B), CD105 (C), CD73 (D)) and CD45 (E), a pan leukocyte marker, which is not expressed on ASCs. Black histograms represent isotype controls.

S1b Tri-lineage differentiation

The tri-lineage differentiation potential of ASCs was induced by culture in differentiation media for 14 days, followed by the verification of differentiation by standard staining methods (Oil Red O, Alcian Blue, and Alizarin staining, respectively). Media were changed every three to four days.

Adipogenic differentiation was induced in adipogenic medium containing high glucose content (4.5 g/L), insulin (1.74 µM, Novo Nordisk), dexamethasone (0.1 µM, Ratiopharm), isobutyl-methylxanthin (0.5 mM, Sigma), indomethacine (200 µM, Fluka), and 10% FBS. Oil Red O (Sigma) staining revealed the accumulation of lipid droplets in intracellular vacuoles indicating adipogenic differentiation. The chondrogenic differentiation of ASCs was induced in chondrogenic medium containing ascorbic acid (50 nM; Merck), insulin (6.25 µg/mL, Novo Nordisk), transforming growth factor β (10 ng/mL, Peprotech) and 1% FBS. The chondrogenic phenotype was assessed by Alcian Blue 8GX staining (Fluka). Osteogenic differentiation of ASCs was induced in osteogenic medium containing ascorbic acid (50 µM; Merck), glycerophosphate (10 mM, Sigma), dexamethasone (1 µM, Ratiopharm), recombinant bone morphogenic protein-2 (100 ng/mL, Immunotools, Friesoythe) and 15% FBS. After 14 days of incubation, the osteogenic phenotype was assessed by staining according to Alizarin Red S staining (Fluka).

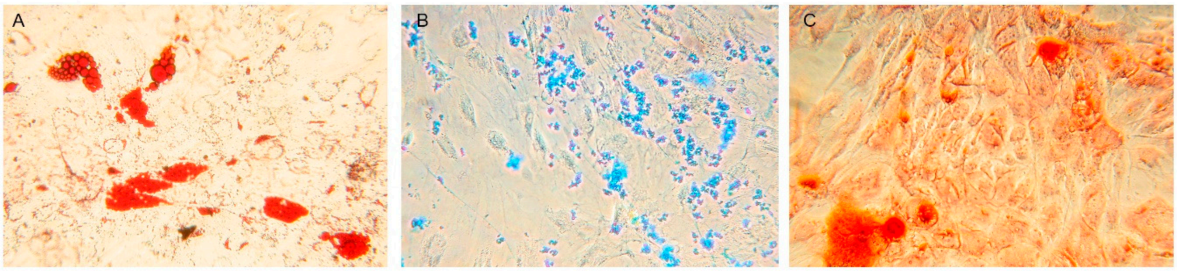

Fig. S1b: Tri-lineage differentiation. Differentiation into adipocytes, chondrocytes and osteoblasts was induced by adipogenic (**A**), chondrogenic (B) and osteogenic (**C**) medium for 14 days. After 14 days of incubation in differentiation medium, cultures were stained with Oil Red O (**A**), Alcian Blue 8GX (**B**) or Alizarin Red S (**C**).

S2 *Quantitative PCR*

We performed a single-step RNA isolation protocol using Nucleozol (Macherey-Nagel, Düren, Germany) to extract whole RNA from cultured ASCs. Then, cDNA was synthesized from isolated RNA for 30 min at 37 °C using 1 µg RNA, 50 µM random hexamers, 1 mM deoxynucleotide-tripheosphate mix, 50 units of reverse transcriptase (Fermentas, St. Leon-Rot, Germany) in 10× PCR buffer, 1 mM β-mercaptoethanol and 5 mM $MgCl_2$. A Hot FIREPol EvaGreen Mix Plus was used (Solis Biodyne, Tartu, Estonia) for the master mix; the primer mix and RNAse-free water were added. Quantitative PCR (qPCR) was carried out in 96-well plates using the following conditions: Twelve minutes at 95 °C for enzyme activation, 15 s at 95 °C for denaturation, 20 s at 63 °C for annealing and 30 s at 72 °C for elongation (40 cycles). Finally, a melting curve analysis was executed. The quantification of the PCR fragments was performed using the ABI Prism® 7900HT Fast Real-Time PCR System with a Sequence Detection System SDS 2.4.1 (Thermo Fisher Scientific, Germany). Relative quantification was assessed by the ΔΔCT method [Pfaffl 2001 [36]], using β-actin as a calibrator, and levels of target gene expression were estimated by $2^{-\Delta\Delta Ct}$. Primer pairs were synthesized by Thermo Fisher Scientific (Germany) and are listed in Table 1.

Table 1. Primer used for qPCR analyses.

Gene	Primer Forward	Primer Reverse	Product Length (bp)	NCBI Reference Sequence
VEGF	CTGTCTAATGCCCTGGAG	TATCGATCGTTCTGTATCAGT	269	NM_001025366.3
IGF2	TCCTCCCTGGACAATCAGAC	AGAAGCACCAGCATCGACTT	243	NM_000612.6
β-actin	ACT GGA ACG GTG AAG GGT GA[illegible]	AGA GAA GTG GGG TGG CTT TT	169	NM_001101

S3 *Additional characterization of SEC-isolated EVs by Western blotting*

The expression of CD44, CD63, and b-actin was examined using Western blotting, as previously described [35]. In brief, EVs or ASCs were lysed using 10x RIPA buffer (Cell Signaling, Frankfurt/M., Germany) and collected. After centrifugation, the pellet was suspended in Laemmli's buffer and heated at 95 °C for 5 min prior to electrophoresis on a 10 % SDS polyacrylamide gel. The protein content was determined by a standard assay and an equal volume of protein was loaded into each lane. The separated proteins were transferred electrophoretically to Immobilon transfer membrane and blocked for 2 h. Membranes were then incubated with monoclonal antibodies against human CD44 or CD63 (both from Immunotools, Friesoythe, Germany, both used 1:250), or b-Actin (GenScript, Leiden, Niederlande, 1:1000), followed by a horseradish peroxidase-conjugated anti-mouse IgG secondary antibody (DAKO Agilent, Germany, No. P0447). For detection, the eECL system (Thermo Fisher Scientific, Germany) was used, protein bands were made visible using the Peqlab Fusion FX system (VWR, Darmstadt, Germany).

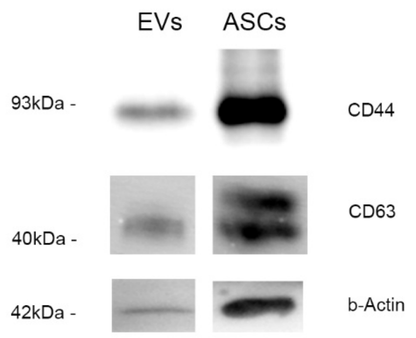

Fig. S2: Additional characterization of SEC-isolated EVs and corresponding ASCs by Western blotting
